# Supplementary material for: Survey on Carbapenem-Resistant Bacteria in Pigs at Slaughter and Comparison with Human Clinical Isolates in Italy
Source: Antibiotics (Basel). 2022 Jun 7;11(6):777. doi: 10.3390/antibiotics11060777 (PMC9219774; doi:10.3390/antibiotics11060777)
Supplement: Supplementary file 1 [file antibiotics-11-00777-s001.zip › antibiotics-1734202-supplementary.pdf]

#File	Nseq	Nres	A	C	G	T	N	%GC
CRE102_S1	225	6438088	1081364	2138874	2136192	1081658	0	66.40%
CRE153_S9	317	6396234	1071728	2118782	2130452	1075272	0	66.43%
CRE295_S1	141	6426083	1068292	2102630	2161004	1094157	0	66.35%
CRE98_S10	124	6415664	1080533	2126693	2133348	1075090	0	66.40%
NEF-156_S	172	7099705	1212066	2332328	2343073	1212238	0	65.85%
NEF23_S7_	201	6436450	1079465	2134215	2139549	1083221	0	66.40%

Min	Q25	Med	Q75	Max	N50	N75	N90	L50
237	934	9658	36103	238923	75398	42989	21385	25
253	4204	11727	25462	223278	38674	20193	10835	47
255	780	20157	66543	328392	102638	62745	33838	19
255	999	18002	69871	561477	126411	75855	37989	13
250	721	6722	53532	340617	123256	75109	33598	18
239	995	10914	47273	377949	79626	49438	23837	23

L75	L90
53	86
105	167
38	59
30	46
36	57
49	76
